# Based on PCA and SSA-LightGBM oil-immersed transformer fault diagnosis method

**DOI:** 10.1371/journal.pone.0314481

**Published:** 2025-02-19

**Authors:** Jizhong Wang, Jianfei Chi, Yeqiang Ding, Haiyan Yao, Qiang Guo

**Affiliations:** 1 State Grid Zhejiang Electric Power Co., Ltd., Hangzhou, China; 2 Hangzhou Electric Power Equipment Manufacturing Co., Ltd., Hangzhou, China; Asia University, TAIWAN

## Abstract

A fault diagnosis method for oil immersed transformers based on principal component analysis and SSA LightGBM is proposed to address the problem of low diagnostic accuracy caused by the complexity of current oil immersed transformer faults. Firstly, data on dissolved gases in oil is collected, and a 17 dimensional fault feature matrix is constructed using the uncoded ratio method. The feature matrix is then standardized to obtain joint features. Secondly, principal component analysis is used for feature fusion to eliminate information redundancy between variables and construct fused features. Finally, a transformer diagnostic model based on SSA-LightGBM was constructed, and the ten fold cross validation method was used to verify the classification ability of the model. The experimental results show that the SSA-LightGBM model proposed in this paper has an average fault diagnosis accuracy of 93.6% after SSA algorithm optimization, which is 3.6% higher than before optimization. At the same time, compared with the GA-LightGBM and GWO-LightGBM fault diagnosis models, SSA-LightGBM has improved the diagnostic accuracy by 8.1% and 5.7% respectively, verifying that this method can effectively improve the fault diagnosis performance of oil immersed transformers and is superior to other similar methods.

## 1 Introduction

Oil-immersed transformer is one of the important equipment in the power system, and its operation status determines whether the power system can operate safely and reliably. However, due to the influence of various factors such as electrical stress, thermal stress, and mechanical stress on the transformer, the transformer oil and organic insulating materials gradually age and decompose, resulting in the production of carbon oxides and a small amount of alkane gas, and their physical properties gradually decline. Therefore, it is particularly critical to make accurate diagnosis of transformer fault types by collecting monitoring data of dissolved gas in transformer oil and combining with appropriate fault diagnosis methods. However, due to the lack of a clear mapping relationship between transformer faults and gas content in oil, extracting transformer fault features is still the main problem of Dissolved Gas Analysis (DGA) technology. At present, although the IEC three-ratio method (the three-ratio method is a modification of the Rogers four-ratio method by the International Electrotechnical Commission (IEC)), the Rogers method and the improved three-ratio method have been formed, the fault boundaries of the above methods are too absolute and the coding is incomplete, so that The accuracy of transformer fault diagnosis results is not high [1, 2].

With the rapid development of artificial intelligence research, some intelligent algorithms have been introduced into transformer fault diagnosis. At present, scholars at home and abroad use DGA technology to integrate with support vector machine (SVM), convolutional neural network (CNN) [3], Self-Organizing Map (SOM) Neural Networks [4], fuzzy theory and other methods to improve the accuracy of fault diagnosis. Literature [5–7] uses a variety of intelligent algorithms Optimizing the penalty factor C and kernel function parameter g of SVM can improve its classification accuracy. However, the support vector machine is proposed for the binary classification problem, and it has shortcomings in multi-classification. References [8–10] use convolutional neural network in transformer fault diagnosis to effectively diagnose internal faults of power transformers, but there are problems that it is easy to fall into a local minimum point and the convergence speed is slow. References [11–14] use fuzzy theory to introduce transformer fault diagnosis and state assessment to solve the ambiguity and uncertainty between fault causes, operating states and fault mechanisms, and to better improve the accuracy of transformer faults. Its experience is processed by means of membership functions, and there will be a problem of "cognitive uncertainty". In recent years, with the continuous development of ensemble learning, many scholars have also applied it to transformer fault diagnosis. Among them, literature [15] screened the fault related features combined with the original DGA gas content features and ratio features, and optimized the lightgbm model parameters. Finally, the fault diagnosis accuracy increased from 88.02% to 90.14%. Reference [16] constructed 16 gas volume ratios as the input fault features of the LightGBM model, and used grid search to optimize the learning rate, tree depth, number of leaf nodes and iterations of the LightGBM model, and the final classification accuracy reached 93.39%. Reference [17] uses LightGBM to establish a 10KV feeder fault prediction model for distribution network. After parameter optimization, the fault accuracy of meteorological, equipment and operating factors all exceeds 90%.

Based on the analysis of the current research status of transformer fault diagnosis mentioned above, it can be concluded that traditional methods for analyzing dissolved gases in oil have limitations and the accuracy of fault diagnosis models can be further improved. In response to the first question, this article adopts the uncoded ratio method when constructing features. The uncoded ratio method is similar to the three ratio method, Rogers ratio method, etc., using the dissolved gas concentration ratio or component proportion in oil as the feature parameter. However, the dimensionality of the constructed features is more extensive, covering a wider range of effective information compared to traditional ratio methods. It has stronger potential for fault feature mining and can further improve the fault diagnosis performance of the model. In response to the second issue, this article adopts a lightweight gradient boosting machine model optimized by sparrow search algorithm as the transformer fault diagnosis model, which achieves a fault diagnosis accuracy of 93.6%. Compared with similar models, the diagnostic accuracy of this method has been improved by 8.1 and 5.7 percentage points, respectively. In summary, this article proposes a fault diagnosis method for oil immersed transformers based on principal component analysis (PCA) and sparrow search algorithm combined with LightGBM (SSA-LightGBM). Firstly, the relationship between different characteristic gases generated during the fault is explored, and a 17 dimensional joint feature is constructed using the uncoded ratio method; Secondly, the Z-score method is used for normalization, and the principal component analysis method is used for feature selection to construct fused features and eliminate redundant features between variable information; Finally, a fault diagnosis model for oil immersed transformers based on SSA LightGBM is constructed using the ten fold cross validation principle.

## 2 Data preprocessing

### 2.1 Preprocessing

The gases dissolved in the oil are mainly H_2_, C_2_H_6_, CH_4_, C_2_H_2_, and C_2_H_4_. When a fault occurs, the transformer fault type is determined by the ratio between the characteristic gases. Currently widely used methods include the Three-Ratio Method [18], CUSUM Method [19], and Rogers Ratio Method [20]. Although these methods are simple to operate, they have defects such as incomplete encoding and absolute fault boundary distinction [21]. The Non-Coding Ratio Method is similar to the Three-Ratio Method and Rogers Ratio Method, using the concentration ratio or component ratio of dissolved gases in oil as feature parameters, but with more dimensions in constructing features. Compared to traditional ratio methods, it covers a wider range of effective information, has stronger potential for fault feature mining, and can further improve the fault diagnosis performance of the model. After a large number of simulation experiments, it is found that:

1. There is no C_2_H_2_ in partial discharge, and there are more CH_4_;
2. When arc discharge in oil, the gas generated is mainly H_2_ and C_2_H_2_, with a small amount of CH_4_ and C_2_H_4_;
3. When the temperature is overheated, the total hydrocarbons of H_2_ will increase significantly, and the total hydrocarbons of transformer insulating oil, including C_2_H_4_, C_2_H_6_, and CH_4_, will increase.

Based on the above rules, this paper adopts the uncoded ratio method to construct 17 kinds of characteristic parameters [22], whose characteristic quantities are described in Table 1.

**Table 1:** Feature quantity and number.

Number	Feature amount	Number	Feature amount
*x*_1_	H_2_	*x*_10_	C_2_H_6_/ TH
*x*_2_	CH_4_	*x*_11_	H_2_/ TH
*x*_3_	C_2_H_4_	*x*_12_	CH_4_/ H_2_
*x*_4_	C_2_H_2_	*x*_13_	C_2_H_2_/C_2_H_6_
*x*_5_	C_2_H_6_	*x*_14_	C_2_H_4_/ C_2_H_6_
*x*_6_	TH	*x*_15_	C_2_H_2_/ C_2_H_4_
*x*_7_	CH_4_/ TH	*x*_16_	H_2_/(H_2_+TH)
*x*_8_	C_2_H_4_/TH	*x*_17_	(CH_4_+ C_2_H_4_)/ TH
*x*_9_	C_2_H_2/_/ TH		

Referring to DL/T 722–2014 "Guidelines for Analysis and Judgment of Dissolved Gas in Transformer Oil", the data labels are divided into: normal state, medium and low temperature thermal fault, medium temperature thermal fault, high temperature thermal fault, high energy discharge, low energy discharge and partial discharge. In this paper, 513 cases of fault gas data are selected to construct a joint characteristic matrix, each row represents a fault sample, as shown in Formula (1):

$$x=[\begin{array}{ccc} x_{1,1} & \cdots & x_{1,17}\\ \vdots & \ddots & \vdots \\ x_{513,1} & \cdots & x_{513,17} \end{array}]$$ (1)

### 2.2 Z-score normalization

In order to eliminate the significant differences in dissolved gas values among different types of oil, it is necessary to normalize or standardize the data. Standardizing data can make the model gradient descent faster, while also making the data distribution more uniform, avoiding bias and instability caused by significant numerical differences between different features. After standardization, the range of values for all features is similar, which is beneficial for the model to converge faster and reduce the differences between features, helping to avoid overfitting problems in the model. Therefore, this article introduces the Z-Score standardization method [23, 24].

Data standardization processing, as shown in Formula (2):

$${x^{*}}_{ij}=\frac{x_{ij}-\mu }{\sigma },(i=1\cdots 513;j=1\cdots 17)$$ (2)

where x*_ij_ is the joint feature after normalization; *μ* is the feature mean; *σ* is the feature standard deviation; *x*_ij_ is the original feature. After the standardized transformation, the average number of each type of fault features is 0, and the standard deviation is 1. The degree of feature concentration and dispersion trend are consistent, so as to eliminate the difference between features. The joint features of transformer fault sample features after the standardized processing are shown in Formula (3):

$$x^{*}=[\begin{array}{ccc} {x^{*}}_{1,1} & \cdots & {x^{*}}_{1,17}\\ \vdots & \ddots & \vdots \\ {x^{*}}_{513,1} & \cdots & {x^{*}}_{513,17} \end{array}]$$ (3)

## 3 PCA-based fusion feature matrix construction

Due to the use of the uncoded ratio method to construct high-dimensional feature data based on dissolved gas concentration ratios or component proportions in oil, some ratio features may have linear correlations with the original features, resulting in information redundancy. At the same time, due to the high dimensionality of the data, the computational load during the diagnostic process will increase exponentially, leading to an increase in model running time and seriously affecting the fault diagnosis results. Therefore, feature fusion methods are needed to reduce the dimensionality of the data. The commonly used methods include Principal Component Analysis (PCA)、Scaled dot-product attention [25] and Self-attention Mechanism [26], etc.

Principal component analysis is a data statistical analysis method. Its idea is to map high-dimensional data to new feature subspaces that are linearly independent of each other, eliminate the correlation between variables, reduce data dimensions, and simplify complex data [27].

Step1: Find the covariance matrix R.

$$R=[\begin{array}{ccc} r_{1,1} & \cdots & r_{1,n}\\ \vdots & \ddots & \vdots \\ r_{n,1} & \cdots & r_{n,n} \end{array}]$$ (4)

where: r_nn_ is the correlation coefficient.

Step 2: Calculate the eigenvalues and eigenvectors of the covariance matrix R.

Eigenvalues y_j_:

$$y_{1}\geq y_{2}\geq \cdots \geq y_{n}$$ (5)

Eigenvectors:

$$a_{1}=[\begin{array}{c} a_{11}\\ a_{21}\\ \cdots \\ a_{n1} \end{array}],a_{2}=[\begin{array}{c} a_{12}\\ a_{22}\\ \cdots \\ a_{n2} \end{array}],\cdots ,a_{n}=[\begin{array}{c} a_{1n}\\ a_{2n}\\ \cdots \\ a_{nn} \end{array}]$$ (6)

Where the largest eigenvalue and the corresponding eigenvector represent the variance and direction of the first principal component, and similarly up to the smallest eigenvalue and the corresponding eigenvector represent the variance and direction of the last principal component.

Step 3: Calculate the cumulative contribution.

$$C=\frac{y_{j}}{\sum_{k=1}^{n}y_{k}}$$ (7)

The number of principal components of the model is selected based on the cumulative variance contribution, and when the contribution reaches a fixed value, m principal components can be selected.

**Fig 1:**
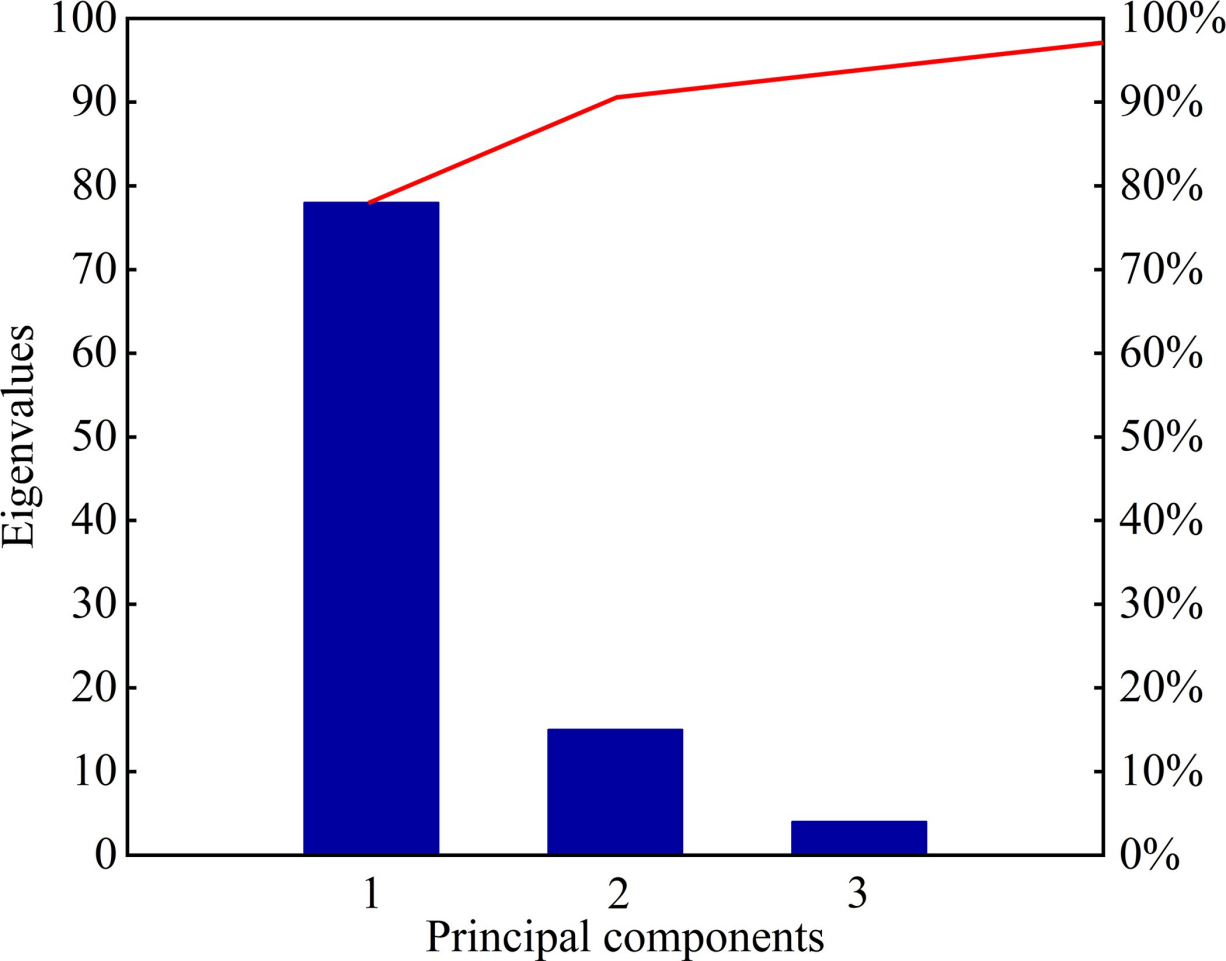
Cumulative contribution rate of the principal components.

In this paper, PCA is used to reduce the dimensionality of the 17-dimensional joint features constructed by the codeless ratio method, and based on the principle of determining the main principal components by the cumulative contribution rate, the cumulative contribution rate of the variance of the first 7 principal elements contains 95% of the information of the principal elements, as shown in Fig 1, so the first 7 principal elements are selected as the fused feature vector matrix for fault diagnosis, which is denoted as:

$$x^{'}=[\begin{array}{ccc} {x^{'}}_{1,1} & \cdots & {x^{'}}_{1,7}\\ \vdots & \ddots & \vdots \\ {x^{'}}_{17,1} & \cdots & {x^{'}}_{17,7} \end{array}]$$ (8)

For the collection of new samples, according to the construction method of the feature vector, construct a 17-dimensional joint feature vector x_new_ = [x_new1_,x_new2_,…,x_new17_], and then project it to the new pivot to obtain the fusion feature *X*new. The projection algorithm is as follows formula:

$$X_{new}=\left[\begin{array}{cccc} x_{new1}, & x_{new2}, & \cdots , & x_{new17} \end{array}\right][\begin{array}{ccc} {x^{'}}_{1,1} & \cdots & {x^{'}}_{1,7}\\ \vdots & \ddots & \vdots \\ {x^{'}}_{17,1} & \cdots & {x^{'}}_{17,7} \end{array}]$$ (9)

**Fig 2:**
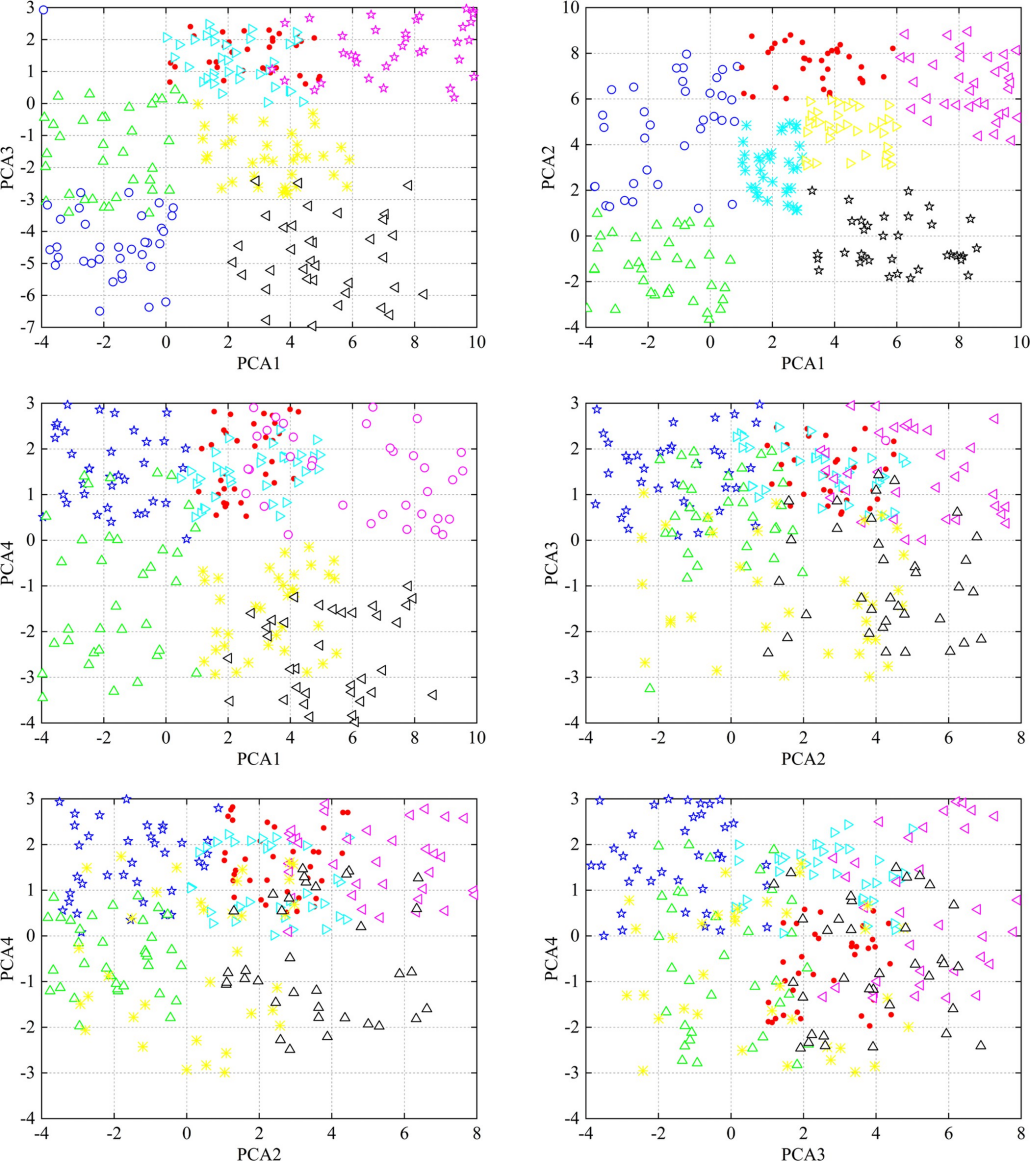
Scatter diagram of different principal elements.

A two-dimensional scatter plot of the different principal elements in the fused feature vector Xnew is presented for verification. From Fig 2, it can be seen that the fused features have good clustering ability.

## 4 Construction of a fault diagnosis model based on SSA-LightGBM

### 4.1 LightGBM algorithm

Light Gradient Boosting Machine (LightGBM) is a decision tree based Boosting algorithm proposed by Microsoft for regression prediction, fault monitoring, classification and feature filtering, with fast training efficiency, low memory consumption and higher accuracy.

The main idea of the LightGBM algorithm is to improve the accuracy of the classifier by multiple iterations based on a decision tree. Assuming that the learner obtained in the previous round is *F*_t-1_(x) and the loss function is *L*(y, F_t-1_(x)) then the objective of the current round needs to find a weak classifier *h*_t_(x) such that the loss function of the current round is minimized, i.e. the minimum loss function is expressed as:

$$h_{t}(x)=\arg_{h\in H}\;\min\sum L(y,F_{t-1}(x)+h_{t}(x))$$ (10)

The approximation of the loss function for the current round is then fitted so that the negative gradient of the loss function can be calculated, and the approximation of the loss function is expressed as:

$$r_{ti}=-\frac{\partial L(y,F_{t-1}(x_{i}))}{\partial F_{t-1}(x_{i})}$$ (11)

Fitting h_t_(x) by the squared difference approximation:

$$h_{t(x)}=\arg_{h\in H}\;\min\sum \left(r_{ti}-h_{t}(x\right){)}^{2}$$ (12)

The resulting strong learners for this round are:

$$F_{t}(x)=h_{t}(x)+F_{t-1}(x)$$ (13)

### 4.2 SSA algorithm

Sparrow search algorithm is a new swarm intelligence algorithm proposed in 2020, which constantly updates individual positions to simulate sparrow foraging and anti predatory behavior.

Initialize the sparrow population and fitness value: suppose there are n sparrows in the population, the population can be expressed as X = [x_1_,x_2_,…,x_n_]^T^, the fitness functions of sparrows are F = [f(x_1_), f(x_2_),…, f(x_n_)], the specific expression is:

$$X=[\begin{array}{ccc} x_{11} & \cdots & x_{1d}\\ \vdots & \ddots & \vdots \\ x_{n1} & \cdots & x_{nd} \end{array}]$$ (14)

$$F=[\begin{array}{c} f([\begin{array}{cccc} x_{11}, & x_{12}, & \cdots , & x_{1d} \end{array}])\\ f([\begin{array}{cccc} x_{21}, & x_{22}, & \cdots , & x_{2d} \end{array}])\\ \cdots \\ f([\begin{array}{cccc} x_{n1}, & x_{n2}, & \cdots , & x_{nd} \end{array}]) \end{array}]$$ (15)

Discoverers account for 10% ~ 20% of the entire sparrow population, and discoverers with better fitness values will preferentially obtain resources in the search process, the location update is shown in Formula (12):

$$x_{i,j}^{t+1}=\{\begin{array}{l} x_{i,j}^{t}\cdot \exp(\frac{-i}{\alpha \cdot iter_{\max}}),R_{2}<ST\\ x_{i.j}^{t}=Q\cdot L,R_{2}>ST \end{array}$$ (16)

In the formula, t represents the current number of iterations; *x*_i,j_^t+1^ represents the position information of the ith sparrow in the jth dimension; α expressed as a uniform random number of (0,1]; iter_max_ represents the maximum number of iterations, *R*_2_ represents the alarm value, and *R*_2_ *ϵ* [0,1]; ST is the safety threshold, and ST *ϵ* [0.5,1]; Q is a random number subject to normal distribution; L is 1×d-dimensional all 1 matrix. When *R*_2_<ST, it indicates that predators do not appear in the area around foraging; when *R*_2_≥ST, it indicates that the predator appears and gives an alarm, all discoverers quickly fly to a safe area.

Followers will conduct local search under the leadership of the discoverer, and followers with better fitness values will give priority to obtain resources in the search process, the location update is shown in Formula (13):

$$x_{i,j}^{t+1}=\{\begin{array}{l} Q\cdot \exp(\frac{x_{worst}^{t}-x_{i,j}^{t}}{t^{2}}),i>\frac{n}{2}\\ x_{p}^{t}+\left|x_{i,j}^{t}-x_{i,j}^{t+1}\right|\cdot A^{+}\cdot L,i\leq \frac{n}{2} \end{array}$$ (17)

In the formula, *x*_p_^t+1^ is the current best position of the producer; *x*_worst_^t^ is the global worst position; A is 1×d-dimensional matrix, each element of the matrix is randomly set to 1 or -1, and A^+^ = A^T^(AA^T^). When i>n/2, it indicates that the ith entrant failed to grab food and needed to go to other areas to look for food; when i≤n/2, it indicates that the entrant is foraging near the optimal individual *x*_p_.

Update the location of the watchman:

$$\{\begin{array}{l} x_{\mathrm{best}}^{t}+\beta \cdot |x_{i,j}^{t}-x_{\mathrm{best}}^{t}|,f_{i}\neq f_{g}\\ x_{\mathrm{best}}^{t}+k(\frac{x_{i,j}^{t}-x_{\mathrm{best}}^{t}}{|f_{i}-f_{w}|+\varepsilon }),f_{i}=f_{g} \end{array}$$ (18)

In the formula, *x*_best_^t^ is the global optimal position; β is a step size control function and follows a normal distribution function with a mean of 0 and a variance of 1; k is the moving direction of sparrows, and k *ϵ* [–1,1]; ε is a constant, avoiding zero denominator; f_i_ is the fitness value of the current individual; *f*_g_ and *f*_w_ are the current global optimal and worst fitness values, respectively.

### 4.3 Model parameter optimization and training test

Because LightGBM model has diagnostic capability under default parameters, but the diagnostic accuracy is low, searching for specific hyperparameters in LightGBM model can further improve the fault accuracy. Where the parameter max_depth represents the depth of the tree, adjusting reasonably to avoid generating too deep trees [28]; feature_leaves represent a subsampling of features; learning rate represents the learning efficiency, when learning rate is set too small, the gradient decreases very slowly, if learning rate is too large, it will cross the optimal value, causing oscillation; subample is used to train weak learners, and it is easy to get too small a value to fit. Table 2 describes the main parameters, the optimization range, and the optimal parameter.

**Table 2:** Key parameters of LightGBM.

Parameter name	Optimization range	Optimal parameter
max_depth	(4,12)	6
feature_leaves	(0,1)	0.7
learning rate	(0,1)	0.814
subsample	(0,1)	0.632

**Fig 3:**
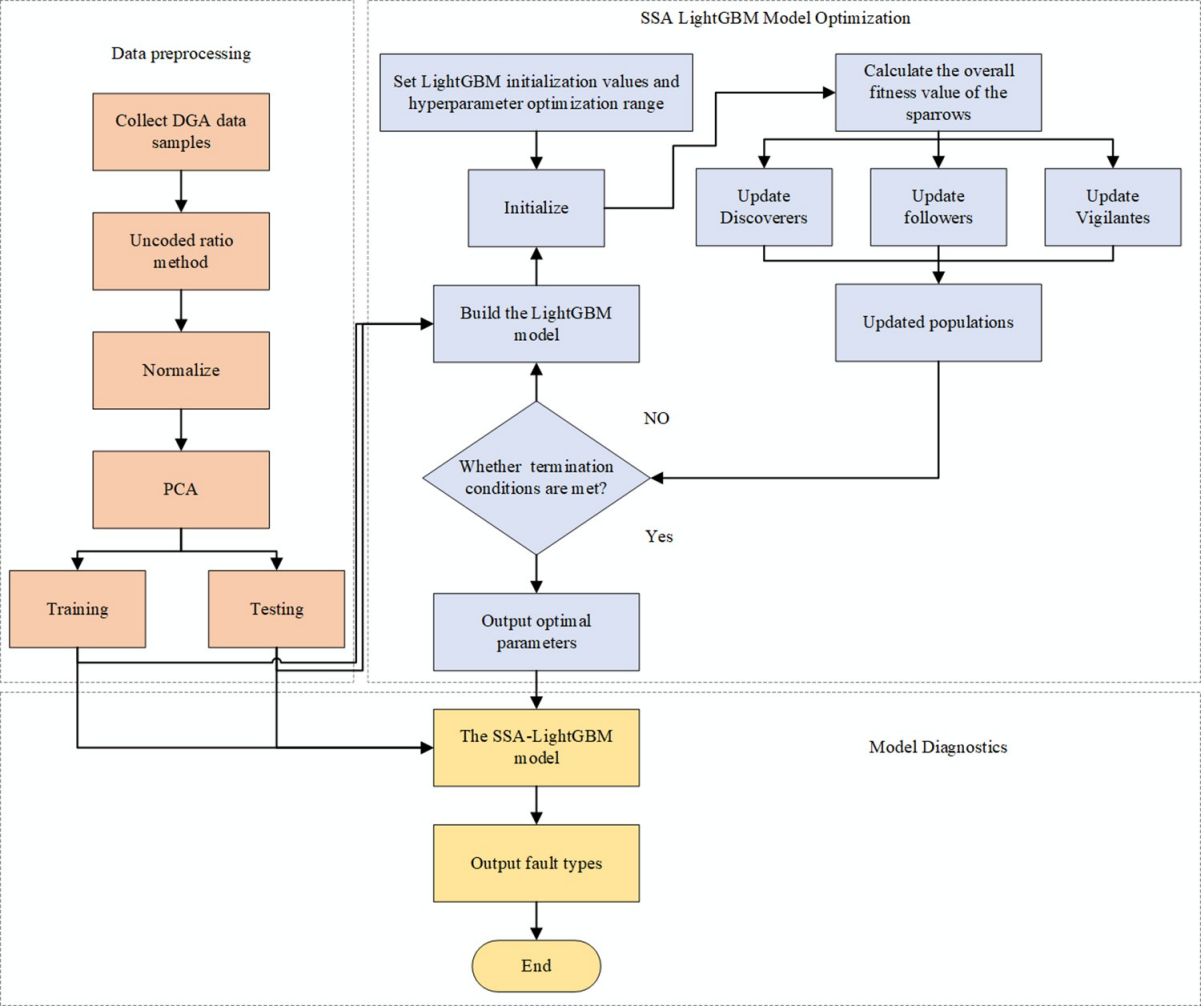
PCA and SSA-LightGBM based transformer fault diagnosis flow chart.

The SSA algorithm is used to optimize the parameters of LightGBM, so as to build the SSA-LightGBM diagnosis model, the diagnosis flow chart is shown in Fig 3.

Step 1: According to the collected data samples, the non coding ratio is used to construct the fault features, and then it is standardized, so as to eliminate the influence of the value between different ratios;

Step 2: Combined with the ten fold cross validation method, the sample data is divided into training set and test set;

Step 3: Set the initial parameters and super parameter optimization range of LightGBM model, and adjust the model parameters using SSA algorithm;

Step 4: Calculate the fitness value of the new position of the sparrow, and update the best and worst fitness values and their positions experienced by the entire sparrow population;

Step 5: Judge whether the number of iterations has been reached, if so, terminate the iteration, store the currently obtained training parameter value as the optimal parameter, otherwise return to Step 3;

Step 6: Input the stored optimal parameters into the LightGBM model, use the test set to test the diagnosis effect of the model, and output the fault classification results.

### 4.4 Comparative analysis of diagnostic results

#### 4.4.1 Analysis of fault type diagnosis results

In this paper, 513 cases of characteristic gases at transformer faults were collected, and the constructed SSA-LightGBM diagnostic model was trained and tested by inputting preset parameters and combining the ten-fold cross-validation principle, and its average fault accuracy was calculated to be 93.7% and the overall fault accuracy was 94.8%, as shown in Table 3.

**Table 3:** Sample diagnostic results and sample distribution.

Working Status	Total number of samples	Number of test samples	Number of correct diagnoses	Accuracy
Normal state	43	13	11	0.856
High temperature thermal fault	45	14	11	0.786
Medium temperature thermal fault	50	15	15	1
Medium and low temperature thermal failure	128	39	38	0.974
Low energy discharge	50	15	15	1
High energy discharge	78	23	23	1
Partial discharge	119	35	33	0.943
Total	513	154	146	0.943

#### 4.4.2 Analysis of multi-model diagnosis results

To verify the superiority of LightGBM diagnostic model, the same fused features are used as input, and the diagnostic results of LightGBM model are compared with KNN [29], SVM [21] and BP [30]. The results are shown in Table 4. The average accuracy of LightGBM model reaches 90.3%, which is better than other models, verifying the superiority of LightGBM model, and after SSA optimizing, the accuracy is improved by 3.4%, which effectively improves the diagnosis accuracy.

**Table 4:** Diagnostic accuracy of different models.

Model Name	Diagnostic accuracy (%)
KNN	77.8%
SVM	74,5%
BP	83.3%
LightGBM	90.3%
SSA-LightGBM	93.7%

In this paper, SSA is used to find the relevant hyperparameters of LightGBM diagnosis model and compare and analyze the optimization results with those of GA and GWO, and the diagnosis results are shown in Table 5. The model diagnosis accuracy of GA-LightGBM, GWO-LightGBM and SSA-LightGBM are 85.6%, 88% and 93.7%, respectively, and SSA-LightGBM LightGBM model has the highest diagnostic accuracy, and all medium-temperature thermal faults, low-energy discharges and high-energy discharges are correctly identified, indicating that the SSA-LightGBM fault diagnosis model constructed in this paper can effectively improve the fault diagnosis accuracy of transformers.

**Table 5:** Multi-model fault diagnosis results.

Models Name	Diagnostic accuracy		
	GA-LightGBM	GWO -LightGBM	SSA-LightGBM
Normal state	0.822	0.759	0.856
High temperature thermal fault	0.774	0.781	0.786
Medium Temperature Thermal fault	0.896	0.959	1
Medium and low temperature thermal failure	0.871	0.921	0.974
Low energy discharge	0.881	0.943	1
High energy discharge	0.876	0.904	1
Partial discharge	0.873	0.895	0.943
Average	0.856	0.880	0.937

#### 4.4.3 Analysis of diagnostic results for different feature quantities

In order to verify the effectiveness of the optimized 7-dimensional fusion features, the experimentally collected data were used to obtain the corresponding fault diagnosis accuracy by IEC triple ratio, Rogers method and no-coding ratio method, and the results are shown in Table 6, indicating that the fusion fault features can dig deeper into the connection between the fault type and the DGA data, which can further improve the accuracy of the fault diagnosis model.

**Table 6:** Average accuracy of different feature quantities.

Characteristic quantity name	Accuracy rate (%)
IEC Three Ratio	87.4%
Rogers	86.9%
Joint Features	88.4%
Integration characteristics	93.7%

## 5 Conclusion

In summary, the method proposed in this article can effectively solve the problem of low diagnostic accuracy caused by the complexity of oil immersed transformer faults, improve the fault diagnosis performance of transformers, and is superior to other methods. The specific innovation points are as follows:

(1) Propose to construct 17 dimensional fault features based on the uncoded ratio method, and deeply explore the intrinsic relationship between dissolved gases in oil and fault types, which is beneficial for extracting transformer fault features.

(2) Propose a PCA based method for dimensionality reduction of fault features, effectively removing invalid and redundant features, while reducing computational complexity and improving diagnostic accuracy after feature extraction.

(3) Propose a LightGBM model optimized based on sparrow search algorithm, and verify its better accuracy compared to other models by comparing simulation results under the same feature input conditions with different models.

Although the method proposed in this article can achieve higher accuracy in transformer fault diagnosis and has certain generalization and adaptability, it is difficult to apply to online fault diagnosis of transformers due to the complexity of the method for collecting dissolved gases in oil. In the future, online diagnostic methods based on multi-source information fusion should be implemented by combining transformer vibration signals and sound signals.

## Supporting information

S1 Table Initial sample. (PDF)
