# Correction: Based on PCA and SSA-LightGBM oil-immersed transformer fault diagnosis method

**DOI:** 10.1371/journal.pone.0337958

**Published:** 2025-12-03

**Authors:** Lizhong Wang, Jianfei Chi, Yeqiang Ding, Haiyan Yao, Qiang Guo

The first author’s name is spelled incorrectly. The correct name is: Lizhong Wang. The correct citation is: Wang L, Chi J, Ding Y, Yao H, Guo Q (2025) Based on PCA and SSA-LightGBM oil-immersed transformer fault diagnosis method. PLoS ONE 20(2): e0314481. https://doi.org/10.1371/journal.pone.0314481.
