# Supplementary material for: Based on PCA and SSA-LightGBM oil-immersed transformer fault diagnosis method
Source: PLoS One. 2025 Feb 19;20(2):e0314481. doi: 10.1371/journal.pone.0314481 (PMC11838915; doi:10.1371/journal.pone.0314481)
Supplement: S1 Table — (PDF) [file pone.0314481.s001.pdf]

S1 Table Initial sample

H2	CH4	C2H6	C2H4	C2H2	Working Status
14.7	3.7	10.5	2.7	0.2	Normal state
7.5	5.7	3.4	2.6	3.2	Normal state
220.0	340.0	42.0	480.0	14.0	Normal state
30.0	110.0	137.0	52.0	22.3	Normal state
80.0	10.0	4.0	1.5	0.0	Normal state
46.1	11.6	33.1	8.5	0.6	Normal state
33.7	3.0	33.2	27.7	2.5	Normal state
46.8	36.9	8.5	7.5	0.3	Normal state
39.2	24.5	18.4	11.4	6.5	Normal state
42.0	33.6	14.8	9.0	0.6	Normal state
13.6	5.3	8.2	29.0	2.1	Normal state
32.0	11.4	8.2	30.6	3.9	Normal state
0.0	0.8	0.5	0.4	0.0	Normal state
48.0	38.4	91.6	5.3	4.8	Normal state
0.0	1.2	1.6	1.1	0.6	Normal state
2.4	1.4	1.0	0.3	0.0	Normal state
14.0	11.0	3.0	22.0	1.0	Normal state
5.8	2.7	1.8	0.8	0.0	Normal state
53.6	17.7	13.2	5.0	0.0	Normal state
110.0	5.0	2.7	3.2	1.7	Normal state
3.9	1.3	1.5	0.8	0.0	Normal state
0.3	0.3	0.0	0.3	0.0	Normal state
33.0	26.0	6.0	5.3	0.2	Normal state
27.2	26.5	1.9	2.8	0.0	Normal state
12.0	12.5	3.4	13.2	0.5	Normal state
2.2	4.4	0.7	0.9	0.0	Normal state
21.6	4.8	3.3	20.6	0.0	Normal state
0.0	0.0	0.0	0.0	0.0	Normal state
0.0	7.3	21.5	18.6	0.0	Normal state
12.3	8.9	14.0	18.2	0.0	Normal state
0.0	1.8	1.0	2.2	0.4	Normal state
0.0	2.7	1.6	6.1	0.7	Normal state
4.9	1.5	4.1	0.9	0.0	Normal state
5.8	2.5	1.8	1.3	0.0	Normal state
0.0	1.4	2.2	0.8	0.0	Normal state
16.7	1.1	0.9	0.6	0.0	Normal state
14.7	3.7	10.5	2.7	0.2	Normal state
132.9	20.4	8.1	0.6	0.2	Normal state
14.7	3.8	10.5	2.7	0.2	Normal state
63.1	16.7	4.3	9.3	10.1	Normal state
14.7	3.7	10.5	2.7	0.2	Normal state
15.0	12.0	5.3	3.2	0.2	Normal state
87.0	41.0	110.1	72.0	1.0	Normal state
172.9	334.1	172.9	812.5	37.7	High temperature thermal fault
172.9	334.1	172.9	812.5	37.7	High temperature thermal fault
172.9	334.1	172.9	812.5	37.7	High temperature thermal fault
172.9	334.1	172.9	812.5	37.7	High temperature thermal fault
172.9	334.1	172.9	812.5	37.7	High temperature thermal fault
172.9	334.1	172.9	812.5	37.7	High temperature thermal fault
172.9	334.1	172.9	812.5	37.7	High temperature thermal fault
172.9	334.1	172.9	812.5	37.7	High temperature thermal fault
172.9	334.1	172.9	812.5	37.7	High temperature thermal fault
172.9	334.1	172.9	812.5	37.7	High temperature thermal fault
172.9	334.1	172.9	812.5	37.7	High temperature thermal fault
172.9	334.1	172.9	812.5	37.7	High temperature thermal fault
172.9	334.1	172.9	812.5	37.7	High temperature thermal fault

172.9	334.1	172.9	812.5	37.7	High temperature thermal fault
172.9	334.1	172.9	812.5	37.7	High temperature thermal fault
172.9	334.1	172.9	812.5	37.7	High temperature thermal fault
172.9	334.1	172.9	812.5	37.7	High temperature thermal fault
172.9	334.1	172.9	812.5	37.7	High temperature thermal fault
172.9	334.1	172.9	812.5	37.7	High temperature thermal fault
172.9	334.1	172.9	812.5	37.7	High temperature thermal fault
172.9	334.1	172.9	812.5	37.7	High temperature thermal fault
172.9	334.1	172.9	812.5	37.7	High temperature thermal fault
172.9	334.1	172.9	812.5	37.7	High temperature thermal fault
172.9	334.1	172.9	812.5	37.7	High temperature thermal fault
172.9	334.1	172.9	812.5	37.7	High temperature thermal fault
172.9	334.1	172.9	812.5	37.7	High temperature thermal fault
172.9	334.1	172.9	812.5	37.7	High temperature thermal fault
172.9	334.1	172.9	812.5	37.7	High temperature thermal fault
172.9	334.1	172.9	812.5	37.7	High temperature thermal fault
172.9	334.1	172.9	812.5	37.7	High temperature thermal fault
172.9	334.1	172.9	812.5	37.7	High temperature thermal fault
172.9	334.1	172.9	812.5	37.7	High temperature thermal fault
172.9	334.1	172.9	812.5	37.7	High temperature thermal fault
172.9	334.1	172.9	812.5	37.7	High temperature thermal fault
172.9	334.1	172.9	812.5	37.7	High temperature thermal fault
172.9	334.1	172.9	812.5	37.7	High temperature thermal fault
172.9	334.1	172.9	812.5	37.7	High temperature thermal fault
172.9	334.1	172.9	812.5	37.7	High temperature thermal fault
172.9	334.1	172.9	812.5	37.7	High temperature thermal fault
172.9	334.1	172.9	812.5	37.7	High temperature thermal fault
172.9	334.1	172.9	812.5	37.7	High temperature thermal fault
172.9	334.1	172.9	812.5	37.7	High temperature thermal fault
172.9	334.1	172.9	812.5	37.7	High temperature thermal fault
172.9	334.1	172.9	812.5	37.7	High temperature thermal fault
172.9	334.1	172.9	812.5	37.7	High temperature thermal fault
36.2	12.6	4.4	15.5	0.0	Medium temperature thermal fault
105.0	169.4	59.1	347.6	1.8	Medium temperature thermal fault
45.0	168.0	82.0	330.0	3.1	Medium temperature thermal fault
39.4	40.6	17.2	51.4	4.3	Medium temperature thermal fault
62.0	112.0	36.0	148.0	0.0	Medium temperature thermal fault
8.4	28.7	13.0	105.0	2.1	Medium temperature thermal fault
206.0	198.9	74.0	612.7	15.1	Medium temperature thermal fault
220.0	340.0	42.0	480.0	14.0	Medium temperature thermal fault
130.0	440.0	180.0	730.0	0.0	Medium temperature thermal fault
170.0	320.0	53.0	520.0	3.2	Medium temperature thermal fault
50.0	90.0	18.0	260.0	5.9	Medium temperature thermal fault
677.0	457.0	45.0	410.0	12.0	Medium temperature thermal fault
207.0	416.0	347.0	1345.0	20.0	Medium temperature thermal fault
4020.0	15850.0	9057.0	22621.0	535.0	Medium temperature thermal fault
56.0	100.0	51.0	153.0	2.3	Medium temperature thermal fault
160.0	223.0	45.0	495.0	11.0	Medium temperature thermal fault
42.0	98.0	156.0	610.0	0.0	Medium temperature thermal fault
766.0	990.0	115.0	99.0	0.0	Medium temperature thermal fault
51.0	71.0	15.0	87.0	0.0	Medium temperature thermal fault
32.0	60.6	34.5	151.3	0.0	Medium temperature thermal fault
18.0	113.0	76.0	294.0	0.0	Medium temperature thermal fault
318.0	57.0	42.0	219.0	0.0	Medium temperature thermal fault
99.4	50.6	19.0	70.6	0.4	Medium temperature thermal fault
27.4	63.2	32.7	137.8	0.5	Medium temperature thermal fault
63.3	58.1	8.7	60.1	0.0	Medium temperature thermal fault
3303.0	5652.0	7057.0	7633.0	0.0	Medium temperature thermal fault

200.0	1000.0	400.0	3000.0	30.0	Medium temperature thermal fault
330.0	2000.0	1600.0	5100.0	30.0	Medium temperature thermal fault
14.7	610.0	151.0	917.0	0.0	Medium temperature thermal fault
2000.0	8850.0	1500.0	9400.0	200.0	Medium temperature thermal fault
1680.0	7190.0	3700.0	19300.0	140.0	Medium temperature thermal fault
0.0	300.0	6000.0	16900.0	50.0	Medium temperature thermal fault
0.0	260.0	160.0	800.0	0.0	Medium temperature thermal fault
18.0	250.4	172.8	707.9	0.0	Medium temperature thermal fault
890.0	729.0	246.0	1816.0	22.0	Medium temperature thermal fault
17.0	50.6	10.1	3.6	0.0	Medium temperature thermal fault
477.0	566.0	239.0	734.0	116.0	Medium temperature thermal fault
56.4	42.7	167.0	1413.0	17.1	Medium temperature thermal fault
74.8	482.0	297.0	1409.0	7.5	Medium temperature thermal fault
220.0	502.0	178.0	1017.0	7.8	Medium temperature thermal fault
6.9	80.6	47.4	351.0	4.9	Medium temperature thermal fault
131.0	253.0	97.0	357.0	2.4	Medium temperature thermal fault
113.0	140.0	111.0	405.0	28.4	Medium temperature thermal fault
7.8	111.0	129.0	701.0	2.8	Medium temperature thermal fault
58.0	103.0	51.0	251.0	6.0	Medium temperature thermal fault
143.0	305.0	78.0	721.0	2.0	Medium temperature thermal fault
170.0	300.0	44.0	580.0	8.9	Medium temperature thermal fault
180.0	340.0	52.0	6303.0	10.0	Medium temperature thermal fault
17.0	21.0	11.0	145.0	4.1	Medium temperature thermal fault
90.0	160.0	54.0	330.0	29.0	Medium temperature thermal fault
44.6	63.2	20.1	129.0	0.7	Medium and low temperature thermal failure
246.0	432.0	141.0	674.0	7.2	Medium and low temperature thermal failure
56.0	144.0	36.0	164.0	1.8	Medium and low temperature thermal failure
69.0	212.0	135.0	692.0	3.9	Medium and low temperature thermal failure
181.0	316.0	115.0	593.0	2.0	Medium and low temperature thermal failure
170.0	330.0	77.0	430.0	13.0	Medium and low temperature thermal failure
76.0	202.0	275.0	3216.0	16.0	Medium and low temperature thermal failure
87.0	500.0	26.0	520.0	9.0	Medium and low temperature thermal failure
279.0	487.0	109.0	708.0	4.4	Medium and low temperature thermal failure
1852.0	1004.0	2447.0	2557.0	696.0	Medium and low temperature thermal failure
94.0	195.0	14.0	153.0	15.0	Medium and low temperature thermal failure
210.0	1300.0	300.0	1500.0	13.0	Medium and low temperature thermal failure
229.0	1110.0	170.0	713.0	0.6	Medium and low temperature thermal failure
19.0	47.0	6.9	61.5	0.0	Medium and low temperature thermal failure
37.0	130.0	91.0	430.0	2.9	Medium and low temperature thermal failure
35.0	30.0	10.0	93.0	7.1	Medium and low temperature thermal failure
336.0	419.0	105.0	1074.0	21.0	Medium and low temperature thermal failure
56.0	27.0	20.0	108.0	6.0	Medium and low temperature thermal failure
78.0	161.0	86.0	353.0	10.0	Medium and low temperature thermal failure
22.3	7.8	12.4	95.7	1.4	Medium and low temperature thermal failure
35.1	50.6	16.1	93.0	1.1	Medium and low temperature thermal failure
56.0	99.9	51.4	24.8	2.3	Medium and low temperature thermal failure
63.0	149.6	57.5	276.0	0.0	Medium and low temperature thermal failure
68.0	99.2	35.9	202.9	0.0	Medium and low temperature thermal failure
236.0	410.2	159.0	817.3	3.5	Medium and low temperature thermal failure
293.0	134.0	52.0	239.0	2.9	Medium and low temperature thermal failure
2.0	16.7	3.9	76.5	0.9	Medium and low temperature thermal failure
20.4	17.0	5.7	52.8	4.0	Medium and low temperature thermal failure
4.1	20.8	7.0	67.6	0.5	Medium and low temperature thermal failure
17.2	21.4	5.4	54.9	1.1	Medium and low temperature thermal failure
17.3	21.7	5.8	52.3	2.8	Medium and low temperature thermal failure
11.3	21.8	11.2	53.1	2.5	Medium and low temperature thermal failure
14.1	26.9	7.4	48.5	3.2	Medium and low temperature thermal failure
2.0	29.1	11.3	57.7	0.0	Medium and low temperature thermal failure

27.4	35.0	5.7	29.4	2.4	Medium and low temperature thermal failure
30.1	39.0	4.6	26.1	0.2	Medium and low temperature thermal failure
63.0	109.2	39.1	211.5	0.0	Medium and low temperature thermal failure
53.0	191.0	77.0	548.0	8.4	Medium and low temperature thermal failure
97.9	103.3	31.6	131.3	19.7	Medium and low temperature thermal failure
148.0	239.0	113.0	878.0	4.0	Medium and low temperature thermal failure
80.0	243.0	274.2	1347.7	8.4	Medium and low temperature thermal failure
82.0	161.0	79.0	406.0	8.9	Medium and low temperature thermal failure
82.0	81.0	33.0	224.0	5.4	Medium and low temperature thermal failure
88.0	83.0	28.0	196.0	7.7	Medium and low temperature thermal failure
180.0	97.0	34.0	271.0	8.0	Medium and low temperature thermal failure
92.0	62.0	35.0	280.0	11.0	Medium and low temperature thermal failure
87.0	87.0	26.0	218.0	7.5	Medium and low temperature thermal failure
121.0	69.0	32.0	268.0	8.0	Medium and low temperature thermal failure
19.6	320.7	279.2	574.7	0.0	Medium and low temperature thermal failure
457.0	79.0	54.0	190.0	2.4	Medium and low temperature thermal failure
97.0	405.0	190.0	583.0	34.0	Medium and low temperature thermal failure
19.6	320.7	574.7	279.2	0.0	Medium and low temperature thermal failure
166.6	28.3	6.7	12.4	0.3	Medium and low temperature thermal failure
143.0	305.0	721.0	78.0	2.0	Medium and low temperature thermal failure
147.0	766.0	506.0	2078.0	3.1	Medium and low temperature thermal failure
26.0	88.3	46.0	196.0	0.0	Medium and low temperature thermal failure
172.9	334.0	172.0	812.5	37.7	Medium and low temperature thermal failure
56.0	99.9	51.4	248.8	2.3	Medium and low temperature thermal failure
46.0	98.0	41.3	26.3	0.0	Medium and low temperature thermal failure
60.0	139.0	21.0	430.0	4.6	Medium and low temperature thermal failure
304.0	443.5	110.9	946.0	56.1	Medium and low temperature thermal failure
105.0	210.0	77.0	675.0	10.0	Medium and low temperature thermal failure
300.0	125.0	80.0	140.0	3.0	Medium and low temperature thermal failure
231.4	357.6	138.9	467.1	0.5	Medium and low temperature thermal failure
25.0	85.0	9.6	178.0	1.4	Medium and low temperature thermal failure
29.9	34.6	5.6	70.0	1.2	Medium and low temperature thermal failure
12.0	290.0	144.0	355.0	0.0	Medium and low temperature thermal failure
83.0	35.0	4.9	86.0	5.1	Medium and low temperature thermal failure
4.9	21.0	15.0	69.3	3.8	Medium and low temperature thermal failure
140.0	173.0	45.0	17.0	0.0	Medium and low temperature thermal failure
11.0	31.0	26.0	52.0	0.0	Medium and low temperature thermal failure
9.0	16.7	9.9	85.3	3.4	Medium and low temperature thermal failure
5.5	9.6	3.9	50.0	1.5	Medium and low temperature thermal failure
23.0	61.0	14.0	153.0	4.4	Medium and low temperature thermal failure
44.2	101.0	13.0	11.8	0.5	Medium and low temperature thermal failure
18.0	20.0	6.0	12.0	2.0	Medium and low temperature thermal failure
181.0	262.0	210.0	528.0	0.0	Medium and low temperature thermal failure
160.0	130.0	33.0	96.0	0.0	Medium and low temperature thermal failure
4.3	193.0	118.0	125.0	0.0	Medium and low temperature thermal failure
170.0	320.0	53.0	520.0	3.2	Medium and low temperature thermal failure
27.0	90.0	42.0	63.0	0.2	Medium and low temperature thermal failure
9259.0	8397.0	26782.0	10497.0	63.2	Medium and low temperature thermal failure
120.0	120.0	33.0	84.0	0.6	Medium and low temperature thermal failure
57.0	77.0	19.0	21.0	0.0	Medium and low temperature thermal failure
259.0	863.0	393.0	994.0	6.0	Medium and low temperature thermal failure
93.0	58.0	43.0	37.0	0.0	Medium and low temperature thermal failure
166.6	28.3	6.7	12.4	0.3	Medium and low temperature thermal failure
38.2	31.0	7.9	22.9	0.0	Medium and low temperature thermal failure
1.0	43.8	26.8	28.4	0.0	Medium and low temperature thermal failure
15.3	22.2	17.8	44.7	0.0	Medium and low temperature thermal failure
7.8	30.4	17.4	43.4	1.0	Medium and low temperature thermal failure
32.8	44.3	10.9	12.1	0.0	Medium and low temperature thermal failure

40.3	25.1	18.6	16.0	0.0	Medium and low temperature thermal failure
33.6	33.6	9.2	23.5	0.2	Medium and low temperature thermal failure
24.0	27.8	24.4	30.0	0.0	Medium and low temperature thermal failure
13.1	44.7	91.5	213.3	4.2	Medium and low temperature thermal failure
70.2	246.6	171.5	371.6	0.0	Medium and low temperature thermal failure
66.0	201.5	220.0	99.8	0.0	Medium and low temperature thermal failure
46.0	98.0	41.3	26.3	0.0	Medium and low temperature thermal failure
86.0	137.0	48.0	230.0	1.1	Medium and low temperature thermal failure
120.0	160.0	55.0	300.0	1.4	Medium and low temperature thermal failure
4.9	10.1	62.1	22.2	0.6	Medium and low temperature thermal failure
40.3	25.1	18.6	16.0	0.0	Medium and low temperature thermal failure
35.4	51.2	8.0	5.5	0.0	Medium and low temperature thermal failure
98.0	155.0	54.0	300.0	1.5	Medium and low temperature thermal failure
12.2	40.5	18.9	28.4	0.1	Medium and low temperature thermal failure
181.0	262.0	41.0	28.0	0.0	Medium and low temperature thermal failure
4.3	193.0	118.0	128.0	0.0	Medium and low temperature thermal failure
93.0	58.0	43.0	37.0	0.0	Medium and low temperature thermal failure
98.5	555.0	200.0	1636.0	8.3	Medium and low temperature thermal failure
300.0	490.0	180.0	360.0	95.0	Medium and low temperature thermal failure
27.0	90.0	42.0	63.0	0.2	Medium and low temperature thermal failure
111.0	559.0	243.0	707.0	0.0	Medium and low temperature thermal failure
679.0	4992.0	1823.0	3671.0	0.0	Medium and low temperature thermal failure
22.2	50.7	25.1	56.9	0.0	Medium and low temperature thermal failure
78.0	70.1	294.8	635.8	12.2	Medium and low temperature thermal failure
181.0	262.0	210.0	528.0	37.7	Medium and low temperature thermal failure
0.0	434.0	226.0	387.0	0.0	Medium and low temperature thermal failure
4.3	193.0	118.0	125.0	0.0	Medium and low temperature thermal failure
23.5	61.3	45.2	98.0	1.0	Medium and low temperature thermal failure
24.2	261.1	196.1	201.4	0.9	Medium and low temperature thermal failure
93.5	36.7	11.2	69.5	1.1	Medium and low temperature thermal failure
96.0	41.0	12.8	82.5	0.6	Medium and low temperature thermal failure
220.0	230.0	57.0	110.0	7.0	Medium and low temperature thermal failure
97.0	229.0	136.0	227.0	1.0	Medium and low temperature thermal failure
910.0	1592.0	801.0	932.0	0.0	Medium and low temperature thermal failure
20.0	41.9	20.2	44.2	0.4	Medium and low temperature thermal failure
24.0	27.8	24.4	30.0	0.0	Medium and low temperature thermal failure
345.0	112.3	27.5	51.5	58.8	Low energy discharge
565.0	93.0	34.0	47.0	0.0	Low energy discharge
550.0	53.0	34.0	20.0	0.0	Low energy discharge
115.9	75.0	14.7	25.3	6.8	Low energy discharge
78.0	161.0	86.0	353.0	10.0	Low energy discharge
54.0	7.0	7.4	8.6	5.4	Low energy discharge
19.0	10.0	7.9	9.5	8.5	Low energy discharge
80.0	20.0	6.0	20.0	62.0	Low energy discharge
170.0	24.0	7.0	17.0	54.0	Low energy discharge
4670.0	3500.0	2120.0	5040.0	2560.0	Low energy discharge
1256.0	80.0	62.0	38.0	0.0	Low energy discharge
24.3	16.4	1.7	30.2	27.5	Low energy discharge
58.0	18.9	4.6	8.7	9.9	Low energy discharge
87.3	6.5	5.2	1.1	0.0	Low energy discharge
34.6	18.2	14.4	17.3	15.5	Low energy discharge
613944.0	10.8	4.2	10.4	13.2	Low energy discharge
45.0	11.1	12.7	2.7	28.5	Low energy discharge
90.0	5.3	2.0	2.7	0.0	Low energy discharge
52.5	32.9	9.7	79.3	9.8	Low energy discharge
6.0	0.5	0.3	1.9	7.4	Low energy discharge
63.0	0.3	0.6	3.6	16.8	Low energy discharge
22.5	8.5	1.2	7.3	8.4	Low energy discharge

31.0	6.6	4.7	19.0	67.0	Low energy discharge
3.3	1.1	0.9	3.1	3.3	Low energy discharge
45.0	15.0	1.0	19.0	18.0	Low energy discharge
316.0	36.1	37.3	109.0	440.0	Low energy discharge
24.2	3.2	3.7	16.6	101.0	Low energy discharge
35.2	2.6	1.9	5.3	18.3	Low energy discharge
4.1	3.5	0.7	1.2	5.2	Low energy discharge
34.0	60.0	11.0	123.0	6.0	Low energy discharge
96.0	13.7	3.8	35.6	5.4	Low energy discharge
12.0	14.6	7.2	14.4	1.0	Low energy discharge
11.9	12.4	5.9	13.6	1.0	Low energy discharge
46.7	8.2	1.8	5.1	4.1	Low energy discharge
53.9	8.3	1.1	6.0	7.1	Low energy discharge
51.1	7.1	1.7	5.9	9.2	Low energy discharge
49.1	12.2	0.3	3.9	4.8	Low energy discharge
912.5	130.0	6.9	32.1	307.6	Low energy discharge
176.0	205.9	47.7	75.7	68.7	Low energy discharge
100.0	94.0	22.0	304.0	118.0	Low energy discharge
29.0	26.3	1.8	27.0	82.4	Low energy discharge
650.0	53.0	34.0	20.0	0.0	Low energy discharge
1565.0	98.0	35.0	48.0	0.0	Low energy discharge
97.8	15.9	2.7	8.1	24.4	Low energy discharge
66.0	8.3	8.2	9.2	8.2	Low energy discharge
116.5	623.1	416.2	1683.5	2.9	Low energy discharge
59.0	10.4	4.0	10.0	12.7	Low energy discharge
68.0	8.0	3.0	8.0	11.0	Low energy discharge
85.9	7.0	4.5	2.6	0.0	Low energy discharge
84.3	8.1	7.2	0.4	0.0	Low energy discharge
83.7	8.1	5.2	3.0	0.0	Partial discharge
200.0	50.0	40.0	50.0	6.0	Partial discharge
18.6	18.0	6.7	55.5	1.2	Partial discharge
34.7	28.0	20.0	16.0	1.3	Partial discharge
44.5	50.4	4.6	0.0	0.5	Partial discharge
30.1	17.1	2.2	5.5	5.2	Partial discharge
675.1	426.9	81.4	993.0	347.6	Partial discharge
61.8	43.0	9.1	113.2	70.6	Partial discharge
201.7	48.9	17.0	122.0	132.3	Partial discharge
50.0	38.8	10.6	108.6	75.3	Partial discharge
61.0	30.1	13.0	73.1	16.1	Partial discharge
30.0	62.0	60.0	460.0	3.4	Partial discharge
5.2	8.4	6.6	19.0	18.0	Partial discharge
30.0	7.4	8.5	1.8	19.0	Partial discharge
980.0	73.0	58.0	12.0	0.0	Partial discharge
345.0	112.3	27.5	51.5	58.8	Partial discharge
78.0	28.0	13.0	29.0	110.0	Partial discharge
345.0	112.3	27.5	51.5	345.0	Partial discharge
29.0	26.3	1.8	27.0	29.0	Partial discharge
30.1	17.1	2.2	5.5	30.1	Partial discharge
137.0	16.0	18.0	20.2	16.5	Partial discharge
173.0	55.8	14.0	26.0	30.5	Partial discharge
60.0	35.0	4.8	12.0	11.5	Partial discharge
980.0	73.0	58.0	12.0	0.0	Partial discharge
650.0	53.0	34.0	20.0	0.0	Partial discharge
1565.0	93.0	34.0	47.0	0.0	Partial discharge
260.0	8.0	2.5	2.0	0.0	Partial discharge
2587.2	7.9	4.7	1.4	0.0	Partial discharge
180.9	0.6	0.2	0.2	0.0	Partial discharge
56.0	61.0	75.0	32.0	31.0	Partial discharge

73.0	12.3	3.3	27.1	47.9	Partial discharge
560.0	0.0	0.6	0.7	0.0	Partial discharge
1565.0	93.0	34.0	47.0	0.1	Partial discharge
1308.0	125.0	112.0	6.0	0.0	Partial discharge
625.0	49.0	9.0	7.0	0.6	Partial discharge
228.8	20.9	11.7	6.3	3.3	Partial discharge
1313.2	130.2	117.2	11.2	5.2	Partial discharge
118.4	272.4	115.4	666.4	42.4	Partial discharge
628.4	49.2	9.1	8.0	5.0	Partial discharge
227.1	18.4	12.6	5.3	3.2	Partial discharge
120.9	111.9	436.7	83.6	2.3	Partial discharge
1655.6	94.6	35.1	47.5	4.2	Partial discharge
654.7	57.7	37.0	20.9	3.8	Partial discharge
225.5	17.6	8.4	3.0	0.0	Partial discharge
120.0	109.0	435.0	80.0	0.0	Partial discharge
1651.0	90.0	33.0	45.0	2.0	Partial discharge
654.0	55.0	34.0	20.0	0.0	Partial discharge
2587.2	7.9	4.7	1.4	0.0	Partial discharge
485.0	35.0	29.0	6.0	0.0	Partial discharge
480.5	37.0	30.0	6.0	0.0	Partial discharge
102.0	108.0	70.0	41.0	0.0	Partial discharge
98.0	252.0	95.0	646.0	22.0	Partial discharge
334.0	39.9	47.8	5.4	247.6	Partial discharge
217.5	40.0	4.9	51.8	67.5	Partial discharge
1678.0	652.9	80.7	1005.9	419.1	Partial discharge
673.6	423.5	77.5	988.9	344.4	Partial discharge
60.0	40.0	6.9	110.0	70.0	Partial discharge
200.0	48.0	14.0	117.0	131.0	Partial discharge
46.0	37.2	8.3	107.0	71.9	Partial discharge
59.0	28.0	9.0	70.0	15.0	Partial discharge
335.0	67.0	18.0	143.0	170.0	Partial discharge
42.1	28.0	10.0	51.0	5.8	Partial discharge
5405.0	561.0	5.0	70.0	128.0	Partial discharge
44.3	17.3	3.6	23.3	10.4	Partial discharge
189.0	157.0	17.0	62.0	7.4	Partial discharge
260.0	130.0	26.0	84.0	92.0	Partial discharge
980.0	570.0	37.0	480.0	54.0	Partial discharge
940.0	440.0	3.0	530.0	460.0	Partial discharge
420.0	200.0	14.0	230.0	180.0	Partial discharge
60.0	40.0	10.0	110.0	70.0	Partial discharge
707.0	115.0	11.0	55.0	81.0	Partial discharge
57.0	10.5	1.3	13.6	17.7	Partial discharge
44.1	24.6	6.7	24.1	0.5	Partial discharge
20.4	17.2	1.8	24.7	36.0	Partial discharge
30.4	8.1	0.7	16.0	44.7	Partial discharge
17.0	13.8	3.1	39.6	26.6	Partial discharge
62.3	11.2	1.9	10.0	14.6	Partial discharge
45.0	8.1	1.1	18.9	27.0	Partial discharge
32.6	15.5	5.0	38.7	8.3	Partial discharge
44.8	17.5	3.6	23.6	10.5	Partial discharge
23.0	12.0	10.1	12.0	61.8	Partial discharge
2590.2	12.3	7.2	4.2	4.5	Partial discharge
486.4	35.8	33.1	7.5	1.9	Partial discharge
485.3	41.6	31.9	10.3	2.6	Partial discharge
107.0	110.6	71.5	44.6	3.1	Partial discharge
101.5	255.7	98.3	646.6	24.5	Partial discharge
335.4	42.1	48.8	8.4	248.8	Partial discharge
221.8	43.1	8.5	52.0	71.6	Partial discharge

1681.3	653.2	84.7	1010.4	419.8	Partial discharge
139.0	52.2	6.8	62.8	9.6	Partial discharge
117.0	5.8	15.9	7.0	12.8	Partial discharge
421.0	135.0	27.7	351.0	374.0	Partial discharge
71.6	20.2	2.7	34.6	44.2	Partial discharge
730.0	750.0	190.0	1300.0	7.0	Partial discharge
43.0	36.0	41.0	47.0	13.0	Partial discharge
100.0	94.0	22.0	304.0	118.0	Partial discharge
49.0	41.6	25.1	124.0	15.7	Partial discharge
21.0	7.6	4.7	96.3	26.7	Partial discharge
65.0	26.1	10.1	41.6	57.8	Partial discharge
64.0	22.0	20.7	51.7	95.1	Partial discharge
150.0	80.0	235.0	105.0	35.0	Partial discharge
294.0	27.4	5.6	52.6	338.0	Partial discharge
437.0	27.2	22.5	34.9	30.2	Partial discharge
2054.0	370.0	26.0	535.0	805.0	Partial discharge
136.0	140.0	59.0	582.0	140.0	Partial discharge
4430.0	941.0	76.0	2475.0	55585.0	Partial discharge
102.0	62.0	5.0	63.0	73.0	Partial discharge
23.0	12.0	12.0	10.0	61.0	Partial discharge
55.8	37.2	15.6	15.0	1.8	Partial discharge
279.0	41.0	9.7	42.0	34.0	Partial discharge
82.3	14.1	3.1	2.9	0.0	Partial discharge
152.6	22.9	4.9	5.1	0.0	Partial discharge
313.9	33.6	7.6	7.9	0.0	Partial discharge
525.7	71.1	19.8	7.9	0.0	Partial discharge
723.8	113.8	33.3	15.6	0.0	Partial discharge
846.7	142.2	40.6	21.5	0.0	Partial discharge
996.3	211.6	56.3	37.6	1.9	Partial discharge
545.2	82.3	22.8	20.5	0.0	Partial discharge
9.1	2.1	0.0	0.0	0.0	Partial discharge
63.0	11.3	2.2	2.0	0.0	High energy discharge
255.0	48.0	8.3	33.0	29.8	High energy discharge
218.0	195.0	35.0	217.0	33.0	High energy discharge
34.5	21.9	3.2	45.0	19.6	High energy discharge
51.2	37.6	5.1	52.8	51.6	High energy discharge
106.0	24.0	4.0	28.0	37.0	High energy discharge
138.8	52.2	6.8	62.8	9.6	High energy discharge
162.0	35.0	5.6	30.0	44.0	High energy discharge
263.0	628.0	16.8	46.0	855.4	High energy discharge
170.0	24.0	7.0	17.0	54.0	High energy discharge
991.0	91.0	106.0	8.0	389.0	High energy discharge
446.0	93.0	7.0	145.0	637.0	High energy discharge
158.0	71.0	1.3	146.0	685.0	High energy discharge
127.0	107.0	11.0	154.0	244.0	High energy discharge
300.0	240.0	14.0	160.0	140.0	High energy discharge
130.0	98.0	7.0	56.0	65.0	High energy discharge
35.0	25.0	0.0	23.0	22.0	High energy discharge
2251.0	2159.0	887.0	6055.0	1849.0	High energy discharge
198.0	39.0	7.1	27.0	22.5	High energy discharge
200.8	59.5	41.0	40.5	150.8	High energy discharge
1500.0	865.2	99.7	1421.3	662.8	High energy discharge
31.8	25.0	8.2	12.9	5.7	High energy discharge
29.3	24.9	7.3	11.4	7.5	High energy discharge
63.5	7.5	1.9	18.0	6.5	High energy discharge
370.0	119.0	0.0	84.0	199.0	High energy discharge
138.5	68.4	0.0	193.6	137.0	High energy discharge
123.0	51.3	15.6	16.0	42.0	High energy discharge

46.0	11.0	4.0	46.0	71.0	High energy discharge
4.2	11.8	0.0	2.7	9.1	High energy discharge
1000.0	200.0	30.0	400.0	1000.0	High energy discharge
119.0	25.0	12.0	55.0	84.0	High energy discharge
466.5	148.8	13.1	266.4	511.5	High energy discharge
550.4	70.3	9.4	136.7	100.8	High energy discharge
179.0	413.0	27.0	544.0	473.0	High energy discharge
250.0	63.0	2.8	66.0	120.0	High energy discharge
190.0	450.0	240.0	850.0	0.6	High energy discharge
29.0	7.5	5.5	14.8	5.4	High energy discharge
6.3	7.9	7.0	3.0	11.1	High energy discharge
413.0	6.8	40.4	4.6	8.2	High energy discharge
23.4	14.3	7.1	9.7	3.4	High energy discharge
410.0	120.0	18.0	71.0	250.0	High energy discharge
443.0	85.2	9.5	102.9	173.9	High energy discharge
44.4	17.4	3.7	23.3	10.4	High energy discharge
201.0	36.0	6.0	32.3	47.2	High energy discharge
44.1	13.6	2.4	20.1	20.2	High energy discharge
57.0	13.0	0.1	11.0	12.0	High energy discharge
50.1	16.0	4.7	45.4	12.3	High energy discharge
56.0	13.0	2.4	22.0	22.1	High energy discharge
121.0	14.0	3.0	29.0	74.0	High energy discharge
130.4	2.4	7.2	1.8	4.4	High energy discharge
300.4	42.1	3.3	43.2	64.7	High energy discharge
685.0	102.0	9.0	170.0	367.0	High energy discharge
174.0	24.0	9.3	29.3	64.4	High energy discharge
48.2	1.4	4.0	22.5	23.8	High energy discharge
49.8	8.4	2.2	19.4	20.3	High energy discharge
45.7	9.1	2.5	19.5	23.2	High energy discharge
26.9	16.9	3.1	39.4	13.7	High energy discharge
39.2	9.4	2.7	22.9	25.7	High energy discharge
43.7	17.0	2.1	26.2	10.9	High energy discharge
207.1	21.1	4.3	67.3	141.9	High energy discharge
44.4	8.0	1.7	19.3	26.7	High energy discharge
79.0	18.0	7.0	71.0	8.0	High energy discharge
141.0	131.0	13.0	139.0	19.0	High energy discharge
101.0	57.0	13.0	114.0	12.0	High energy discharge
41.1	18.2	2.3	32.2	6.1	High energy discharge
151.0	26.8	36.9	44.6	59.8	High energy discharge
154.3	32.5	24.9	42.4	68.8	High energy discharge
32.4	5.5	1.4	12.6	13.2	High energy discharge
42.0	62.0	5.0	63.0	73.0	High energy discharge
201.0	107.0	19.0	137.0	160.0	High energy discharge
41.6	25.1	124.0	15.7	206.0	High energy discharge
127.0	107.0	11.0	154.0	224.0	High energy discharge
7.0	5.2	0.0	11.0	37.0	High energy discharge
7.6	96.3	26.7	4.7	135.0	High energy discharge
240.0	28.0	6.0	26.0	85.0	High energy discharge
150.0	46.0	19.0	85.0	61.0	High energy discharge
132.0	43.0	20.0	76.0	51.0	High energy discharge
100.0	32.0	9.0	91.0	25.0	High energy discharge
